# Decoding Hindlimb Movement for a Brain Machine Interface after a Complete Spinal Transection

**DOI:** 10.1371/journal.pone.0052173

**Published:** 2012-12-27

**Authors:** Anitha Manohar, Robert D. Flint, Eric Knudsen, Karen A. Moxon

**Affiliations:** 1 School of Biomedical Engineering Science and Health Systems, Drexel University, Philadelphia, Pennsylvania, United States of America; 2 Department of Neurobiology and Anatomy, Drexel University College of Medicine, Philadelphia, Pennsylvania, United States of America; Centre national de la recherche scientifique, France

## Abstract

Stereotypical locomotor movements can be made without input from the brain after a complete spinal transection. However, the restoration of *functional gait* requires descending modulation of spinal circuits to independently control the movement of each limb. To evaluate whether a brain-machine interface (BMI) could be used to regain conscious control over the hindlimb, rats were trained to press a pedal and the encoding of hindlimb movement was assessed using a BMI paradigm. Off-line, information encoded by neurons in the hindlimb sensorimotor cortex was assessed. Next neural population functions, or weighted representations of the neuronal activity, were used to replace the hindlimb movement as a trigger for reward in real-time (on-line decoding) in three conditions: while the animal could still press the pedal, after the pedal was removed and after a complete spinal transection. A novel representation of the motor program was learned when the animals used neural control to achieve water reward (e.g. more information was conveyed faster). After complete spinal transection, the ability of these neurons to convey information was reduced by more than 40%. However, this BMI representation was relearned over time despite a persistent reduction in the neuronal firing rate during the task. Therefore, neural control is a general feature of the motor cortex, not restricted to forelimb movements, and can be regained after spinal injury.

## Introduction

The brain machine interface (BMI) has great potential to restore functional movement after severe injury including spinal cord injury [1], [2], [3], [4], [5], [6], [7], [8]. In this approach the modulations of cortical neurons are used to decode movement related signals in real-time which can then be used as a control signal to restore movement of the affected limb [9], [10] or replace the movement by actuating an external device [4], [11], [12], [13]. Moreover, studies using the BMI experimental paradigms have greatly improved our understanding of how neurons encode for forelimb movement [4], [12], [14], [15], [16], [17], [18]. However, less is known about neural encoding for hindlimb movements or the possibility of using BMI to restore control of the hindlimbs for restoration of hindlimb functions.

Studies examining the neural encoding of hindlimb movement have focused mainly on changes in gait patterns during stereotypic locomotion on a treadmill. Early work by Drew and colleagues [19] showed that pyramidal cells in the hindlimb motor cortex of the cat are involved in the extension and flexion of the limb as the animal steps over an object. Moreover, hindlimb pyramidal cells are involved in gait modifications [20], [21], including the necessary postural adjustments [22] and forelimb/hindlimb coordination produced during changes in gait [23]. In the context of a BMI application, the adaptation to a cortex controlled BMI has been studied in rats during locomotion on the treadmill [24] and during squatting and standing in monkeys [25]. However, control over flexion and extension will be necessary to maintain balance, navigate over varying terrain and avoid obstacles during real-world applications after a spinal cord injury. Therefore, in the work presented here, we examined neural signals that control hindlimb movement, the impact of introducing a BMI on the representation of that signal and the effect of a complete spinal transection. Based on the work from forelimb/arm studies [12], [26], [27], [28], we hypothesized that the encoding of hindlimb movement would be different when explicit behavioral control was replaced by neural control using the brain machine interface. Further changes in encoding were expected after spinal cord injury (SCI).

To test this, we trained rats to press and release a pedal with their hindlimb in response to an audible cue for a reward. We simultaneously recorded populations of single neurons from arrays of microwires chronically implanted in the hindlimb sensorimotor cortex (HL SMC). First, using offline analysis, we measured the information encoded by neurons about the kinematic parameters of movement (offline decoding). Next we used neuronal population functions, weighted representations of the neuronal activity, to replace the hindlimb movement as a trigger for the water reward in real-time (online decoding) and reassessed the ability of the neurons to encode for the motor program when the animals could still press the pedal, after the pedal was removed and after a complete spinal transection. Our results show that during neural control, more information about the motor program is encoded faster than during behavior control. After a complete spinal transection that deafferents the motor cortex and damages the axons of many of these cells, the information about the motor program is initially lost but is eventually regained to levels achieved during behavioral control.

## Results

**Figure 1:**
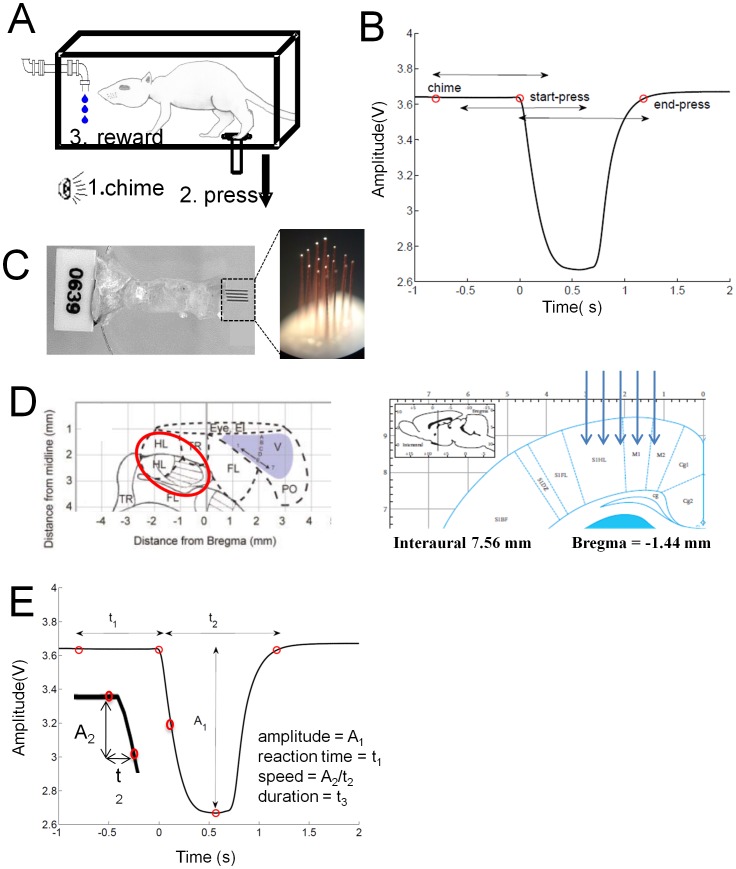
Pedal press task for skilled hindlimb movements. Rats were trained to press a floor-mounted pedal with their hindlimb. (**A**) Schematic of the skilled hindlimb task. (1) After a randomized period of 3–5 seconds, the chime was sounded, cueing (2) the press and release, which, if completed within three seconds of chime onset, led to (3) a water reward. (**B**) Trace of the position sensor indicating the position of the hindlimb during a single trial of the task (black line). The events chime, start-press, and end-press are marked with circles and the arrows indicate the preparation, initiation and movement windows, as defined in the text. (**C**) 16 channel stainless steel microwire array with wires of diameter 50 µm, row/column spacing of 350 µm and insulated with Teflon except at the tip. (**D**) *Left Panel:* Adapted schematic of the rat cortex showing somatotopic representations [56]. Oval indicates target for array implantation in the hindlimb sensorimotor region. *Right Panel:* Coronal view (schematic) of the rat cortex from Paxinos and Watson 2005, arrows indicating the target for the microelectrodes (**E**) Four kinematic parameters were measured from the position sensor to describe the behavior of the animal during the task: reaction time, amplitude, peak velocity and duration.

Single neuron activity was recorded from six animals trained to press and release a pedal using their hindlimb in response to an auditory conditioning stimulus for a water reward (Figure 1A). During *behaviour control* mode (BC), when the animals were rewarded for an appropriate press (see Methods), 14 recordings were made from the 6 animals, with an average of 36±19 cells (mean ± std) recorded per day, 495 cells total. During *neural control* mode (NC), when the animals were rewarded based on their neural activity during the task (see Methods) a total of 17 recordings were made with an average of 40±13 cells (mean ± std) per recording day, 689 cells total.

As expected [4], [12], [24], there were differences in the animal’s press movements during BC mode compared to NC mode (Figure 1F). There were no differences in the reaction time or peak velocity of the animal’s behavior between BC and NC mode. However, both the duration of press (Mann Whitney U test, U = 5.4×10^5^, z = −8.615, p<0.001) and the amplitude of the press (Mann Whitney U test, U = 4.93×10^5^, z = −11.983, p<0.001) were shorter during NC mode than BC mode, suggesting differences between the behavior of the animal under NC mode compared to BC mode (Figure 1F). Therefore, the animals made smaller, quicker movements during NC mode than during BC mode.

Despite these differences in movement during the task, there were no differences in performance (ability to acquire a reward) between BC mode and NC mode. Performance was measured by the number of True Positives (TP) and the number of False Positives (FP). TPs and FPs during BC mode were evaluated by the position of the amplitude sensor while TPs and FPs during NC mode were evaluated by the NPF (see Methods). When performance under NC mode was compared to that during BC mode (Student’s t-test, p>0.05 for both TP and FP) there were no differences, demonstrating that neural activity can be used to replace hindlimb behaviour for a water reward.

### Neuronal Activity in the Hindlimb Sensorimotor Cortex is Modulated during the Task

As expected, a majority of cells recorded from HL SMC modulated their firing rate during the task in both BC and NC mode. Because we expected that different cells would modulate their firing in response to different aspects of the task [19], for each cell, three peri-event time histograms (PETHs) were constructed, each time-locked to one of three different events during the task: the chime cue, the start of press, and the end of press (see Methods). The total number of cells responding to any of the events was 422 out of the total of 495 cells (85%) in the BC mode and 509 out of the 689 cells (74%) recorded in the NC mode.

**Figure 2:**
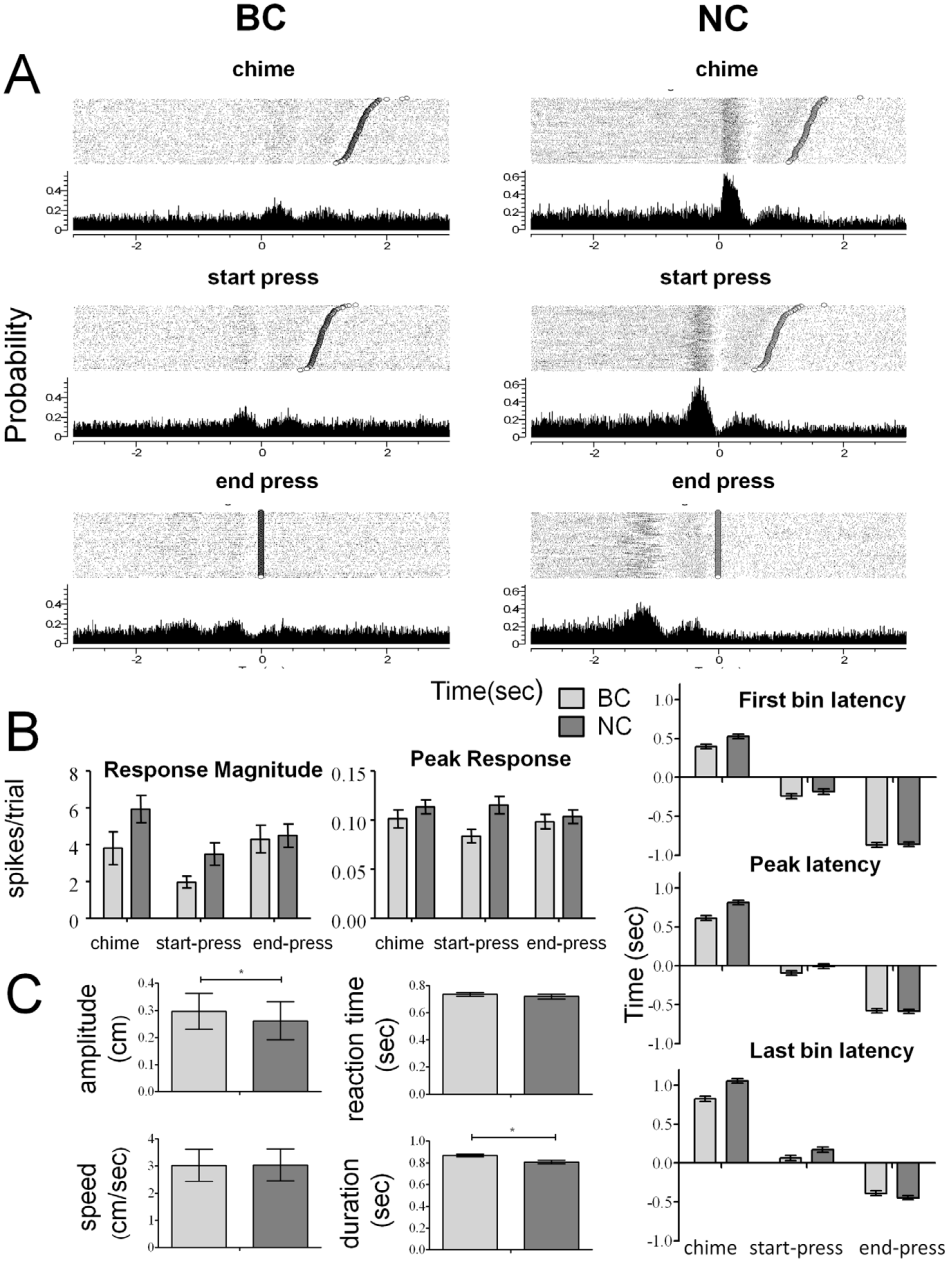
Single neuron activity during pedal press. (**A**) Peri-event time histograms (PETHs) and spike rasters for a representative neuron aligned to different events (chime, start-press, end-press) during the task for BC (left panel) and NC (right panel) mode. The y-axis represents the probability of a spike occurring in a bin of size 5 ms, x-axis represents the time from the event around which the PETH is centered in seconds. Trials are sorted by the duration of the press as measured from the reference event. Open circles in the rasters mark the end of press. (**B**) Differences in neurophysiological parameters (left panel-response magnitude, peak response; right panel- response latencies) between NC and BC mode. See Methods for description of parameters. (**C**) Differences in the four kinematic parameters between modes: behavioral control (BC) and neural control (NC). Asterisks indicate a significant difference.

Although there were no differences in the firing rate across the three different events, there were important differences depending on whether the recordings were done during BC mode or NC mode (Figure 2, two factor ANOVA). The average response magnitude during NC mode was higher than that during BC mode [F (1, 1100) = 4.5547, p<0.05]. In a similar manner, the average peak of the response (5 ms bin), during NC mode was significantly greater than during BC mode [F (1, 1100) = 5.7867, p<0.05]. On the other hand, the latency of the responses did not significantly change across the different experimental modes. The responses of the cells during BC mode (3.39±0.402 spikes/trial) and NC mode (4.88±0.406 spikes/trial) were greater than their responses during passive cutaneous stimulation (0.255 spikes/trial) or during treadmill locomotion (1.49 spikes/trial). This is consistent with earlier work showing that flexion and extension of the limbs to step over an object results in increased activity of HL neurons compared to activity generated during stereotypic treadmill locomotion [19].

### Neural Activity is Correlated to the Parameters of Movement

**Figure 3:**
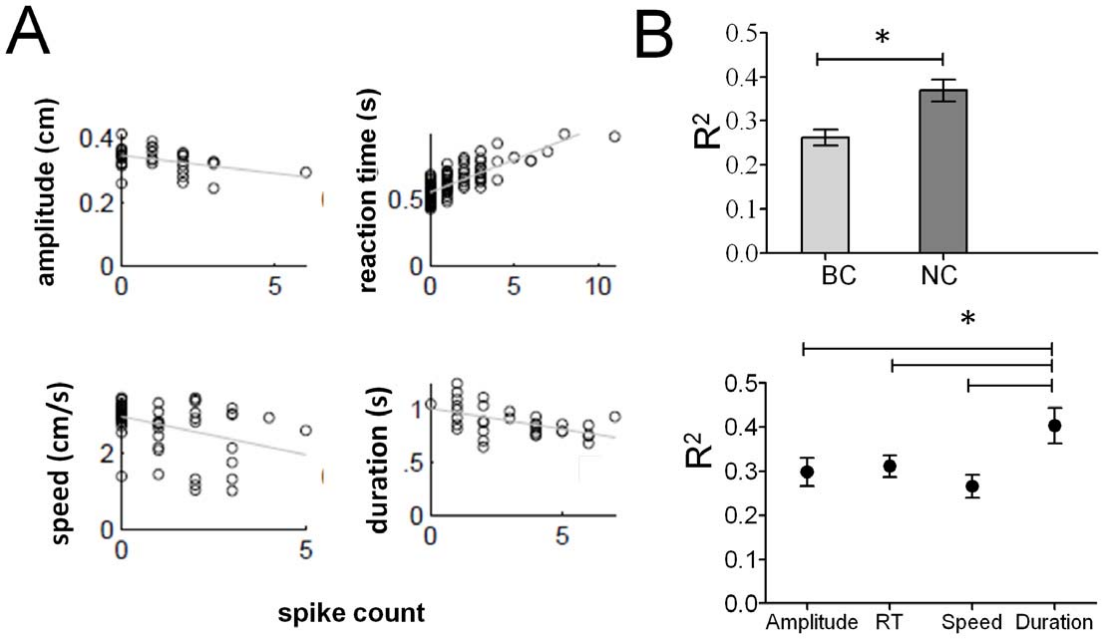
Correlations between the kinematic parameters of movement and the neural activity. (**A**) Magnitude of kinematic parameters (y-axis) plotted as a function of spike count for representative single neurons from the BC mode (x-axis). Each data point corresponds to a single trial and the line is a linear regression. (**B**) **Coefficient of determination** (R^2^) between the kinematic parameters and the numbers of spikes in the response window were different across modes (BC, NC) (top panel) and across kinematic parameters (bottom panel).

The firing rate of most cells was correlated to at least one of the parameters of movement (Figure 3A). In BC mode, the firing rate of 26% of the total 495 cells recorded was significantly correlated to the amplitude of press, 35% to the reaction time, 23% to peak velocity and 36% to the duration of press in at least one of the 3 windows. In NC mode, a similar pattern of activity was found; the firing rate of 30% of the total 689 cells was correlated to amplitude, 40% to reaction time, 18% to peak velocity and 39% to the duration of press in at least one of the 3 windows.

However, firing rates were better correlated during NC mode than BC mode (F(1,112) = 11.503,p<0.001). There was also a significant main effect of the movement parameter (F(3,112) = 3.278, p<0.05). This was due to the fact that the neurons had a significantly higher correlation to duration (R^2^_BC_ = 0.309, R^2^_NC_ = 0.480, Tukey HSD post-hoc test, p<0.05) than to amplitude, reaction time or speed of press (Figure 3B). Therefore, different cells were tuned to a particular movement parameter and they were better tuned during neural control than behavioral control.

### More Information is Generated Faster during Neural Control than Behavioral Control

**Figure 4:**
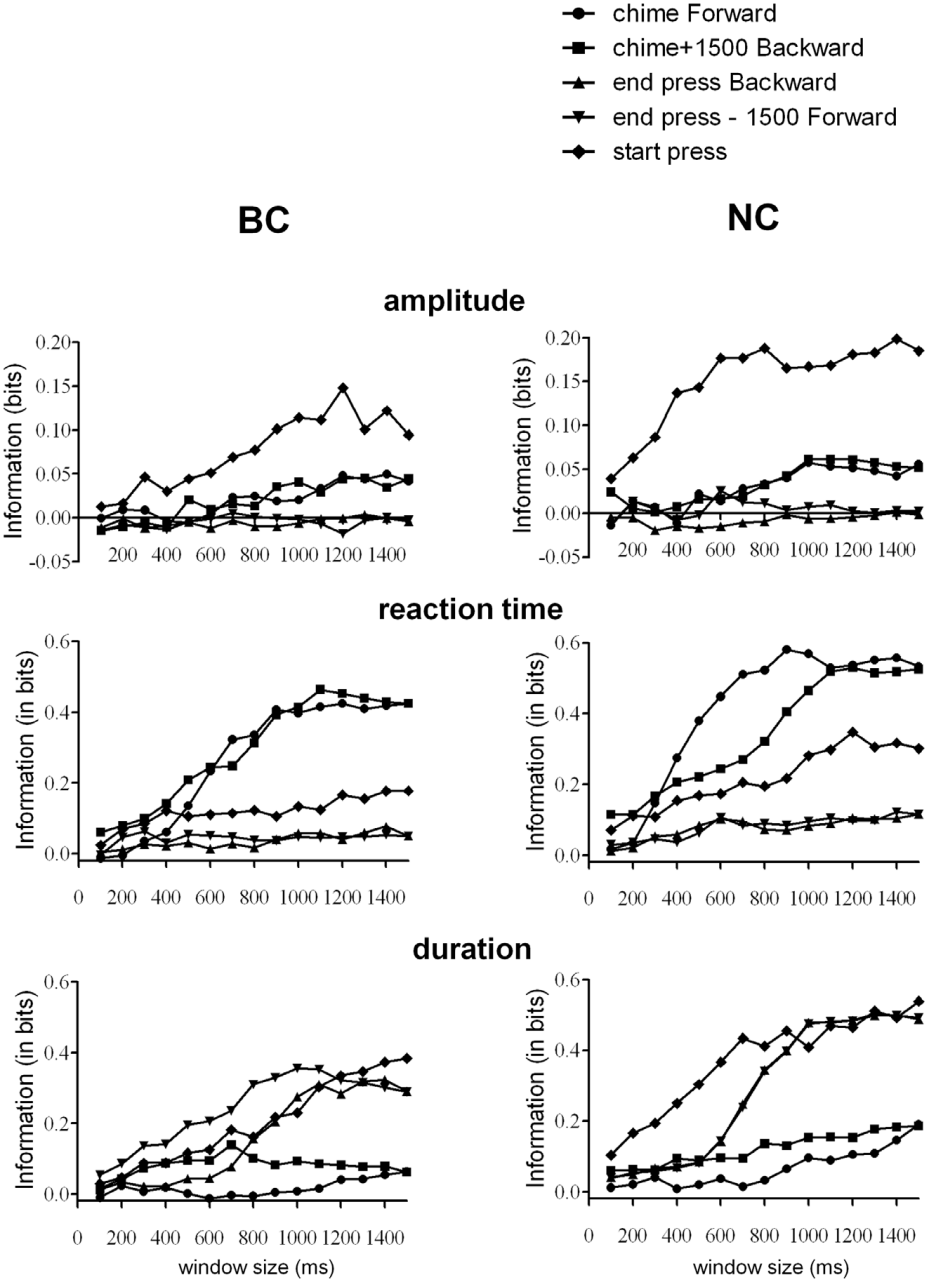
Information encoded by populations of neurons about the kinematics of hindlimb movement. The PETH based classification method was used to quantify the information encoded by simultaneously recorded neurons about each of the movement parameters in two modes of the experiment: BC (left panel) and NC (right panel). Starting from each event (chime, start-press or end press) the window size was incremented by 100 ms. The information is corrected by subtracting the value of the information obtained by shuffling the trials (bootstrapped, see methods). For events chime and end press the windows were incremented from the start and end of the previously used windows and incremented by 100 ms in the forward and backward direction respectively. For the start press event, the window was incremented by 100 ms around the event. The y axis represents the information value in bits, averaged across all recording days. The x axis represents the size of the time window in milliseconds.

There is information in the activity of these cells about hindlimb movements but this activity is substantially different during NC mode compared to BC mode (Figure 4). The information about 3 of the parameters, amplitude, reaction time and duration of the press were successfully decoded and the amount of information was found to be influenced by the time window used to measure it. In the BC mode, the maximum information that could be decoded about the amplitude of press was 0.105±0.03 bits and this was in a window around the start of press. The maximum information about reaction time was decoded from the window after the chime and was equal to 0.424±0.06 bits. The maximum information about the duration of press was decoded from the window around the start of press and was equal to 0.384±0.059 bits. In the NC mode, the maximum information that was decoded about amplitude was 0.185±0.05 bits, about reaction time was 0.533±0.08 bits and about duration was 0.538±0.073 bits, in the same windows respectively.

Importantly, more information was encoded during NC mode compared to BC mode, and the information was obtained faster during NC mode than BC mode. There was a significant main effect of the window length as well as the mode (two factor repeated-measures ANOVA). The information was significantly greater during NC than BC (F(1,91) = 9.0646, p = 0.00337). In the BC mode, the information obtained using any window less than 900 ms was significantly less than the peak information obtained using the entire 1500 ms window (Tukey HSD post hoc test, p<0.001). In the NC mode any window less than 500 ms had significantly less information than the peak value of the information (Tukey HSD post hoc test, p<0.001). There was also a significant interaction effect between the window length and the mode (F(14,1274) = 2.6500, p = .00081), suggesting that the increase in the information occurred differentially in the two modes, and in this case was significantly faster in NC than BC mode. Therefore, the modulation of neuronal firing patterns during NC mode compared to BC mode, identified earlier, results in an increase in the information conveyed by the neurons about the motor program to press.

### PCA for Dimension Reduction is More Efficient during Neural than Behavioral Control

**Figure 5:**
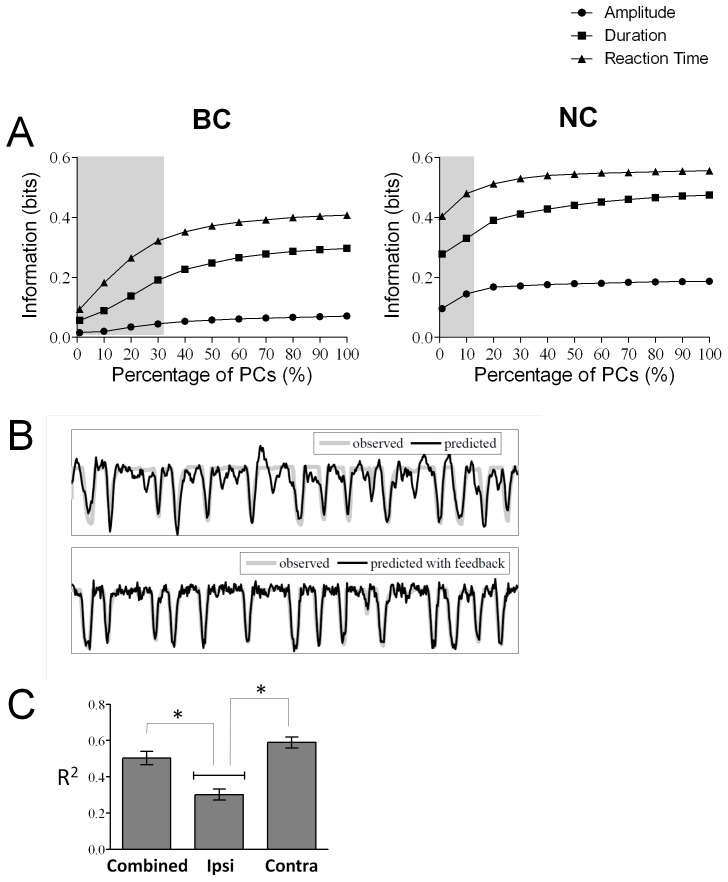
(A) Dimension reduction using principal component analysis. The spike data from the single neurons was used to calculate the principal components (PCs) which were then used to decode information about the press in terms of the behavioral parameters, amplitude, reaction time and duration of press. The information that can be decoded is plotted as a function of the percentage of principal components used to find this information in BC mode (left panel) and NC mode(right panel). Grey rectangle represents minimal percentage of PCs needed such that the amount of information is not different when 100% of PCs are used. (**B**) **Trajectory Reconstruction.** An example of reconstruction of the trajectory of limb movement using Wiener filter from the BC mode. Comparison of the position of the hindlimb (grey) and its prediction using a linear wiener filter (black) without feedback (top panel) and with feedback of the position of the limb (bottom panel). (**C**) Comparing the prediction of trajectory using only the neurons from the contralateral side (brain hemisphere opposite to the limb that was used to press the pedal) to that of the ipsilateral side and when combining neurons from both sides of the cortex. The y-axis represents the R^2^ value between the predicted and actual trajectory for these three conditions- ipsilateral, contralateral and combined.

With an increase in the number of simultaneously recorded neurons to control a brain machine interface or a neuroprosthetic, there arises a need to reduce the high dimensionality of the dataset in order to improve speed and computational efficiency. To test the feasibility of reducing the dimension of neural data [29], [30], principal components (PCs) were used to replace the single neuron spiking data and the information was assessed [29], [30]. While there was no difference in the number of components that account for 90% of the variance in either mode (Students t-test, t(91) = −1.1167, p = 0.25), there was a significant main effect of the first factor, percentage of PCs used (two factor repeated-measures ANOVA, see Methods). In the BC mode, at least 30% of the PCs were required to get values similar to the maximum amount of information (Tukey HSD post hoc test, p<0.001), but in the NC mode only 10% of the PCs were required (p<0.001). There was also a significant effect of the second factor**,** mode of the experiment. The information (F (1, 91) = 9.6580, p = 0.00252) conveyed by 10% of the PCs in NC mode was significantly greater than that during BC mode using 30% of the PCs. There was a significant interaction effect between the percentage of PCs used and the mode (F (10,910) = 3.2819, p = .00035) confirming that the increase in the information by adding more PCs was significantly faster in NC than BC mode (Figure 5A).

### Linear Decoding Algorithms can Decode the Movement Trajectory

Notably, a simple linear filter was able to reconstruct the trajectory of the hindlimb as it pressed the pedal (Figure 5B). Moreover, the information was encoded equally well during NC mode, when the animals is rewarded for its neural activity compared to when the animal is rewarded for its behavior, confirming that the neural activity is continuing to encode for the trajectory of the movement during NC mode.

To test different potential approaches for a clinical application of BMI, the prediction accuracy without feedback was compared to the prediction accuracy with feedback. As expected, the prediction accuracy (measured by the R^2^ value between the actual and predicted signal, see Methods) was better when feedback about the position of the limb from the previous moment in time was incorporated compared to when it was not (One way ANOVA; factor- Method (with feedback, without feedback); F(1,58) = 55.529, p<0.001). Therefore, it is clear that using the position of the limb improves the prediction accuracy; however, it also makes the implementation of a neuroprosthetic more complicated.

Finally, due to the asymmetrical nature of the task, we compared prediction of the limb trajectory using only the neurons from the contralateral (brain hemisphere opposite to the limb that was used to press the pedal) or ipsilateral hemisphere to the prediction when combining neurons from both sides of the cortex (Figure 5C). While there was clearly information about limb trajectory encoded by the neurons ipsilateral to the limb, as expected, the neurons contralateral to the limb encoded more information. In fact, using the information from neurons on both sides did not improve prediction compared to that provided by contralateral neurons. (One-way ANOVA with factor side, F(2,108) = 18.806, p<0.001; Tukey HSD post-hoc, contralateral versus ipsilateral, p<0.001, contralateral versus combined, p = 0.230).

### Neural Activity in the Absence of Movement

**Figure 6:**
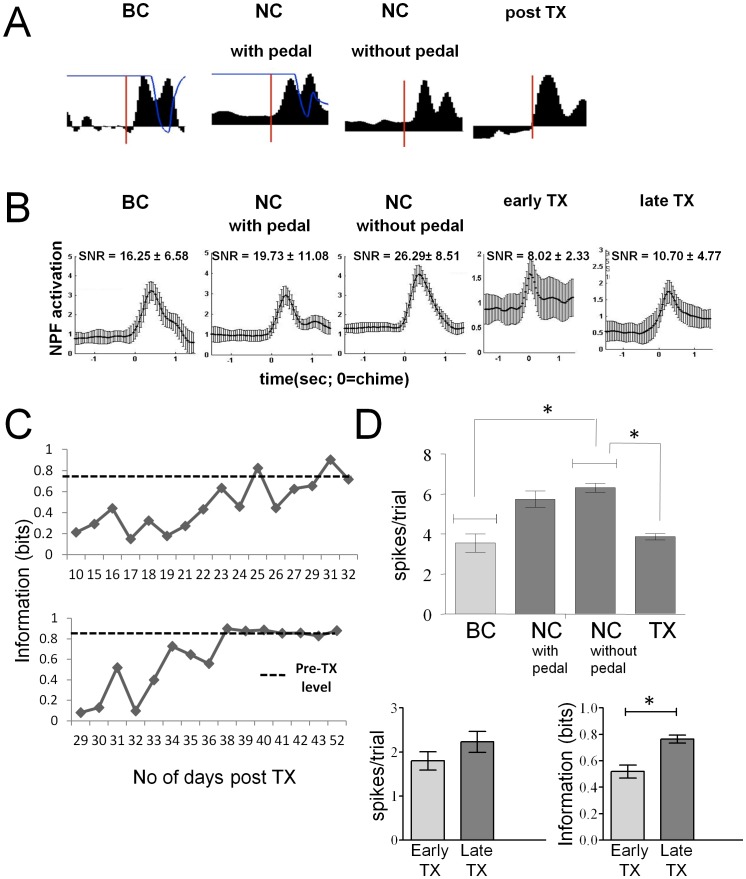
Impact of pedal removal and complete spinal transection on information conveyed by the neurons abut the motor program to press. (**A**) A single trial neural population function plotted as a function of time for BC mode, NC mode with the pedal, NC mode without the pedal and post transection (TX). Thin line, over the population functions for BC and NC with pedal modes, is the output of the amplitude sensor. (**B**) The average neural population functions during a single recording day for BC, NC, NC without pedal, early and late TX stages. Number in each panel refers to the average signal-to-noise ratio (mean ± std). (**C**) Information as a function of absolute time post-TX. *Top panel:* This is the data from one of two animals that were returned to water restriction quickly and reintroduced into the task within 10 days post TX. Despite the quick return to the task, the neurons from this animal also initially conveyed small amount of information about the task but were able to reorganize within a similar time frame to convey information similar to that show during behavioural control (see dotted line). *Bottom panel:* This is the data from one of two animals that were slowly returned to water restriction schedules and introduced into the task 30 days post TX. It took approximately 10 days for the neurons to reorganize to convey information similar to that shown during behaviour control (dotted line). (**D**) *Top panel:* Comparison of the average response magnitudes of the single neurons during the task across the four modes. *Bottom left panel:* Comparison of the response magnitude of the single neurons during the early (first 30%) recording days after complete spinal transection to the late (last 30%). *Bottom right panel:* Comparison of the information about the motor program to press during the early recording days after TX to the late recording days.

To test the impact of removing the pedal on neural encoding, we compared the neuronal responses after the pedal was removed (6 animals, 46 recordings, average of 43±15 cells per recording day, 2014 cells total) to those during behavioural control and to responses during neural control when the animal could still press the pedal. The neural responses were sufficiently similar to those generated during behavioral control to be used on a single trial to trigger a reward to the animal (Figure 6A). In fact there were no differences in the online performance during NC mode without the pedal when compared to that with the pedal (Student’s t-test, p>0.05).

However, there were significant differences in the responsiveness of the neurons across the different modes of recording (Figure 6D top panel, Kruskal-Wallis Test, dependent variable - response magnitude, H(3,5993) = 116.97, p<0.001). When the pedal was removed, the neural activity after the tone increased compared to the activity when the animal could still press (RM_NC without pedal_>RM_BC_, Mann-Whitney U post-hoc, p<0.001). Moreover, the signal-to-noise ratio (SNR) of the response when the pedal was removed was greater than when the animal could press the pedal (Figure 6B). This increase in SNR was due to a reduction in the noise as well as the increase in the magnitude of the signal.

After transection (TX), animals were retested in the task (4 animals were used to obtain 92 recordings, with an average of 43±5 cells per recording day, 4000 cells total). The neuronal activity during the task was reduced to levels originally seen during BC mode (Figure 6D top panel) and was significantly less than during NC mode without pedal (p<0.001). The average number of neurons recorded remained stable but the firing rate of only 65% of the recorded cells were modulated during the task after TX, significantly impacting on-line performance. In fact, on-line performance was initially so low that the animal was given drops of water to maintain its interest in the task despite the fact that its neural activity would not have triggered a reward. This reduction in response magnitude did not improve with time post TX (Figure 6C, bottom left panel) and was initially accompanied by a surprisingly high loss in information about the task during the early TX stage (first 30% of recording days of each animal). The information about the motor program was reduced, well below behavioral control levels (0.75±0.17 bits of information during BC mode compared to 0.51±0.05 bits immediately after SCI, t-test: p<0.05), suggesting a disruption of the network ensemble that encoded information about the press. However, over time, the network re-learned the task and by the late TX stage (last 30% of recording days) the amount of information decoded (0.76±0.03) was significantly increased to levels seen pre-TX (Figure 6C bottom right panel). This increase in the amount of information encoded was accompanied by an increase in on-line performance. The rate of increase was independent of the time post-TX and took approximately 10–15 days. (Figure 6C). This increase in formation was not accompanied by any recovery in the firing rate of the cells.

## Discussion

While the idea of a brain-machine interface for restoration of forelimb movement has been shown to be feasible, those studies were conducted in healthy animals with aid of visual feedback. By translating studies to the hindlimb, the data presented here show for the first time that BMI to control limb function can be used in the absence of visual feedback and after a complete spinal transection. The complete spinal transection deafferents the neurons used to control the BMI and severs the axons of many of the neurons encoding the information. An advantage of this model is that there are no spared fibers to confound the interpretation and our result show that the neurons reorganize to convey sufficient information about the motor program for the animals to gain a reward.

### Role of the Rat Model in Studies of BMI

An important question since the first study of BMI in rats was repeated and extended to non-human primates is whether there is a role for rats in the study of BMI. Despite the fact that the task used here was relatively simple compared to some of the more recent BMI studies in primates [10], [12], [14], [16], [31], the rat model has an important role in BMI studies. First, the data presented here and our earlier work on rat BMI [4], demonstrate remarkable similarity of our results to a broad range of results from primate studies on the upper limbs demonstrating that 1) the impact of BMI in the rat model is similar to the impact of BMI in the primate model and 2) the impact of BMI on the hindlimbs is similar to that on the forelimbs. Studying BMI for restoration of hindlimb function is important because more SCI patients have their hindlimb affected than their forelimbs.

Second, it is well established that nonhuman primates do not navigate complex terrains (uneven terrain, ramps, stairs, etc) in a manner similar to humans and, therefore, it is unlikely, even for studies of hindlimb function in monkeys, that the decoders would be directly translatable to humans. Therefore, the goal of these studies is not to develop decoders that can be directly translated, but rather to understand the principles of BMI. The data presented here demonstrate that the principles of BMI established in primates are also relevant for the rat, making them a good model for the study of neural coding in BMI.

Finally, looking forward to future studies for the role of BMI after injury, the rat model of SCI is one of the best studied animal models of SCI and future work to combine therapies after SCI with BMI would most likely be done in the rat model first. Although still an open question, we expect the impact of BMI and SCI on the ability of neurons within the primary HL-SMC of rats to encode for hindlimb extension and flexion to be a very good indicator of the impact of SCI on encoding in human primary motor cortex for control of a BMI. Of course, this will need to be tested by continuing to make comparisons between the different animal models available and human studies.

### Impact of Neural Control on Single Neuron Responses during Pedal Press

The information decoded about the kinematics of hindlimb press is consistent with the earliest studies of neuronal activity in the HL motor cortex confirming that these neurons are modulated by hindlimb movement [19], [32], [33], [34]. Here, we extend those results to show that neural activity was correlated to the magnitude of the movement parameters during the task. This correlation is similar to previous studies of the modulation of cells recorded from the forelimb motor cortex in response to skilled forelimb reaching tasks [35], [36]. Overall, we observed high correlation values for reaction time and duration of press implying that the cortical resources were largely utilized in encoding the temporal aspects of the task; however, further studies are needed to confirm this. The reason for this could be attributed to the nature of the task, where the animal was trained to press within a certain time window (3 seconds) in order to be rewarded.

Neurons in the HL SMC were also correlated to more than one movement parameter suggesting that cells in the hindlimb cortex may be able to encode for multiple kinematic parameters simultaneously, consistent with studies of encoding of both forelimb/arm and hindlimb movements [12], [23]. Furthermore, certain parameters were better decoded from different windows of time during the task, consistent with forelimb studies [1], [2], [37], [38], [39]. This correlation between the magnitude of the neural response and the magnitude of the movement parameter was functionally relevant because the trajectory of hindlimb movement was well modeled by a linear combination of neural activity, extending the findings of studies on the kinematics of walking patterns [22], [23].

### Decoding Information during Pedal Press

There was an increase in neuronal activity during NC mode compared to BC mode and this increased activity contributed to an increase in information conveyed by the neurons. This increase is consistent with earlier studies using neural control to replace forelimb movements but greater than what would have been expected from those studies [12], [40]. This is likely due to the fact that in our study, the animal did not have independent feedback (e.g. visual) regarding its performance during the task. This is important because learning in the brain machine interface paradigm without visual feedback is required for control of lower limb function. In the forelimb studies, visual tracking during the movements provided the animal with constant feedback about its progress while our animals were required to wait 500 ms after task completion to obtain their only feedback about the neural control, a water reward. This may have made the task more challenging, and hence produced greater cortical modification and the significant increase in information. The degree of cortical modulation has been shown to increase with increased complexity of a learned task [41], [42]. Therefore, our data support earlier studies that increased functional plasticity in the cortex is proportional to the skill required to successfully complete the task, independent of the movement.

The fact that more information could be generated faster during NC mode than BC mode is consistent with the formation of a novel brain state. Practice in a BMI paradigm results in functional reorganization [12], [26] suggesting that the cortex is capable of integrating an external interface into its own representational layout [12], [27], [43], [44]. These differences in neural coding between NC and BC mode clarifies earlier reports and now establishes that differences in the reward schedule alone between behavioral control and neural control are sufficient to induce changes in neuronal firing patterns in contrast to previous studies that relied on visual feedback during the task [40], [45].

Differences in the brain state during neural control compared to behavioral control are further evidenced by the way the information is encoded. The increased average contribution of single neurons to decoding performance and the increased correlation between pairs of neurons contributes to more efficient encoding during neural control as evidenced by the reduction in the number of Principal Components necessary to decode the information. This was true for all of the parameters of kinematic movement tested. Yet, the distribution of the information across the components was still relatively complex during NC mode, requiring at least 12 components to maximize decoding. As a comparison, the sensory information related to whisker stimulation in the rat is contained in the first 3–8 components [30]. Therefore, this is not due to the animal substituting encoding of the kinematics of movement with a simple increase in firing rate for a reward.

It is important to note that during NC mode, when the animal could still press the pedal, there were reductions in the movement, including a smaller amplitude and shorter duration of press compared to BC mode. Similar to studies in primates, this behavioral manifestation suggests that the animal recognizes something different about NC mode compared to BC mode and is likely due to processing of execution-errors when the animal first begins in NC mode (Zacksenhouse et al., 2007). Here, we extend that idea to a task with no visual feedback and to the hindlimb system. The relatively small reduction in movement with concomitant increase in information about the parameters of movement further supports a change in brain state during NC mode compared to BC unrelated to visual feedback.

### Decoding Information in the Absence of Movement

When the pedal is removed, the magnitude of the response and the information about the motor program further increases. Given the limitation of this study, it is not possible to know if the animal is still engaging muscle groups and applying downward pressure despite the removal of the pedal. We expect that, based on our previous work (Chapin, Moxon et al., 1999), that this is unlikely, because the animal learns fairly quickly that they do not have to press to gain a reward. Moreover, the fact that there was a decrease in the variance of the baseline activity (noise) during the post auditory cue window suggests a lack of sensorimotor activity after the pedal was removed which suggests a decrease in muscle activation, but cessation of all activity cannot be confirmed.

It is only after the complete spinal transection that we can be sure that there is no motor output in response to the decoded motor program. Initially, after the transection, the magnitude of the response of neurons to the cue was reduced. This reduction was accompanied by a reduction in information about the motor program to press. The loss in information is likely due to the fact that these neurons become deafferented and the axons of many of these cells are severed. Importantly, despite the fact that the reduction in response magnitude persisted for the duration of recording, information about the motor program to press increased over time, suggesting that the neurons reorganize to convey information about the motor task - they do not simply increase their firing rate to gain a reward. Therefore, neurons in the deafferented HL SMC that have been cut-off from their normal outputs can still organize to convey information about the motor program to press the pedal despite a significant reduction in neuronal activity.

The fact that the HL SMC reorganized to convey information about the motor program to press after a complete TX is consistent with the earliest findings of Fetz and colleagues [45], [46] demonstrating the ability to condition cortical cells to modulate their firing in exchange for a reward, even if the conditioning occurred outside any relevant behavioral context. Importantly, the data presented here show that this can be accomplished even in the injured brain and without simply increasing neuronal firing rate. It is likely that our results are similar to the well established work showing that the healthy motor cortex is capable of modifying its own activation patterns to allow the acquisition and practice of new motor skills [41], [47], [48], [49], [50], [51]. Our data extend this idea to show that this phenomenon is a general property of the motor cortex, not restricted to the forelimb motor cortex, occurs in the absence of visual feedback about the task and after a complete spinal transection. These are important conclusions that advance our understanding of neuroprosthetic control for clinical applications.

## Materials and Methods

### Behavioral Training, Surgery and Electrophysiology

Six adult male Long Evans rats were trained in a hindlimb pedal pressing task. All animal procedures were conducted in accordance with Drexel University Institutional Animal Care and Use Committee-approved protocols, and followed established National Institutes of Health guidelines. Except during recovery from surgery, animal were maintained on a water restriction schedule.

Animals were placed in a behavioral chamber consisting of an 8×12×8 inch clear acrylic cube each day for training (Figure 1A). Near the rear of the chamber, a pedal protrudes from a hole in the floor. Animals were trained to depress and release this pedal with one hindpaw within 3 seconds from the start of an audible chime cue. Successful completion of this task activated a solenoid valve which dispensed approximately 0.1 mL of water after a 500 ms delay. Incorrect trials, either false negative (failure to respond to audible cue within the allotted time) or false positive (incorrectly depressing the pedal in the absence of the audible cue) result in dimming of the house lights for 3 seconds (time-out). Every animal in the study had its own preference of using a particular limb consistently throughout the study. 4 rats used their right limb and 2 used their left. Animals were considered to have achieved proficiency at the task when they completed at least 50 responses per session with a greater than 90% ratio of correct responses to total trials (true positives), together with a less than 10% ratio of inappropriate spontaneous presses to total number of presses (false positives).

Animals that reached proficiency were implanted bilaterally with arrays of 16 microwires into the hindlimb sensorimotor cortex using standard methods in our lab [52] (Figure 1C,D). The electrodes were lowered until they reached the infragranular layer of the cortex, 1.2–1.3 mm from the surface of the brain. Penetration depth was corroborated by the sampled neural activity, when the characteristic large amplitude layer V–VI neurons were present on the majority of electrodes, the array was cemented in place. Throughout the study the activity of neurons from the bilateral arrays were analyzed together.

The experiments were carried out in two distinct control modes based on the factor controlling reward delivery. In the behavior control (BC) mode, the animal was rewarded if the appropriate pedal press behavior was performed in response to the chime cue. In the neural control (NC) mode, the animal was rewarded based on its neural activity during the press. Initially, during NC mode, the animal was still free to press the pedal but eventually, during subsequent recording sessions, the pedal was removed and, finally, the animal was transected and retested in the task. It is important to note that in this task, the animals did not have access to visual feedback indicating their progress towards reward in contrast to studies of forelimb BMI [11], [12], [13], [40] that relied on the animal having some knowledge about its progress during the task under neural control. This is a distinct advantage for the hindlimb task in this study because, presumably, one would not have visual verification of neural control of hindlimb.

Animals were first recorded under BC mode for approximately 4 weeks. Off-line analysis was performed daily to assess the neural performance. In order to do this a neural population function (NPF) was generated by binning the spike times (100 ms bins), and calculated a weighted sum of bin values for all the neurons in the ensemble. The weights used in the NPF were generated using a combined principle component/independent component analysis algorithm [29]. Pedal press/release behavior was typically represented by a peak, either preceded by or followed by a trough, in the smoothed NPF. Therefore, after each chime cue, the NPF value was required to exceed the higher of a set of two thresholds, as well as drop below the lower threshold. This was considered a true positive. During baseline, when the animal was typically sitting quietly, the NPF crossed neither of these thresholds. The thresholds were set such that the true and false positive rates matched the behavioral performance of the animal. If off-line neural performance reached our criteria (90% TP and <10% FP) the animals continued to be tested in BC mode to acquire sufficient baseline data and were then moved to NC mode.

After completion of recordings in BC mode, the animals were run in the NC mode on all subsequent recording days. To get weights for the NPF and to obtain the thresholds, the activity from the previous recording day was used. The data from the previous NC mode recording session were analyzed off-line in a manner similar to that described for off-line analysis during BC mode. During NC mode, the animal was rewarded if the NPF crossed the thresholds derived from the previous day within a time period of 3 seconds after the chime. This process continued regardless of whether there was a pedal. During NC mode and after spinal transection, the weights were derived from off-line analysis of the previous days recording and the thresholds were initially set to the value of the previous day. Because the number of cells recorded could change across days, thresholds were changed to scale them to the peak amplitude of the population function. Since the number of neurons recorded was quite stable, this occurred rarely (on 6.1+/−7.9% of days for the upper threshold and 4.0+/−4.4% for the lower threshold). Once an animal progressed to the next stage (i.e. having the pedal removed) it was not returned to a previous stage.

During both modes, a Multichannel Acquisition Processor (MAP, Plexon Inc., Dallas, TX) was used to simultaneously record from multiple single neurons during each recording session. These signals were high pass filtered with a 400 Hz cut off frequency to extract the action potentials and then digitized at 40 KHz. Discrimination of multiple single units on a single electrode was carried out online before each recording session (Sort Client, Plexon Inc). Pedal pressing activity was transduced using a linear position sensor (P112, Positek, Cheltenham, UK) and sampled at a rate of 200 Hz. The output of this sensor allowed us to continuously track the end-point position of the limb during the task, from which four movement parameters were derived on each trial: reaction time, as well as the amplitude, peak velocity and duration of the press.

The transection procedure was similar to our method in a previous study [53] except here, the animals survived the surgery. Animals were anesthetized with 4% isoflurane, maintained with approximately 1.5% isoflurane, given prophylactic antibiotics (ampicillin 100 mg/Kg) and analgesic (buprenorphine 0.05 mg/Kg) after the surgery. A laminectomy was performed at thoracic level (T8–T9) and the dura was removed with microdissecting scissors. The cord was transected with iridectomy scissors followed by aspiration. A collagen matrix, Vitrogen (Cohesion Technology, Encinitas, CA), was injected into the site of the transection. The lesion was confirmed visually with 20X magnification. The muscle and skin were sutured in layers with 4–0 silk. Animals were given 10 ml of lactated ringer’s solution, placed on a heating pad until they recovered from anesthesia and then returned to their home cage. Animals were given 10 ml of lactated ringers and antibiotics daily for one week post surgery. The animal’s cage was kept on a heating pad and their bladders were expressed 3 times daily until the onset of spontaneous bladder evacuation (7–10 days). Once the animals recovered from surgery (7 days max), they returned to the pre-transection (pre-TX) water restriction levels for the next 5–7 days and then retested.

### Data Analysis

#### Behavioral events

In order to examine the neuronal firing patterns, correlations and information encoded during the task, three behaviorally relevant events were defined, 1) *chime* defined from the time of the auditory cue, 2) *start press* defined by the initial deviation of the amplitude sensor from its baseline position, *3) end press* defined as the completion of the task when the amplitude sensor registered a return to baseline after the press (Figure 1B).

#### Movement parameters

One of the goals in this study was to determine if the motor program of the animals to move the hindlimb could be decoded on a single trial, in real-time. Therefore, as a first approximation, we examined our ability to decode hindlimb movement by defining four movement parameters based on the behavioral events defined above: the *amplitude* of press which is the distance from the baseline position of the pedal to the point of maximum deflection, *reaction time* to press is the time between chime and start-press, *peak velocity* of the press is the peak instantaneous downward velocity and *duration* of press is the time between start-press and end-press. Because we were only decoding gross hindlimb movement, which would be useful for many hindlimb BMI applications, we did not utilize EMGs at this stage of experimentation.

### In Order to Look at How Individual Neurons in the Ensemble Modulated their Activity in Relation to the Task, We Measured their Activity Across Different Modes

#### Peri-event windows

The behavioral events, defined above, were used as reference points to align the neural activity in three windows to allow the identification of the behavioral event that best modulated the neuron’s activity. The *preparation window* started from the chime event and extended 1.5 seconds after chime. The *initiation window* was referenced to the start of press event and extended in 0.75 seconds before and after the event. The *movement* window was referenced to the end press event and started from 1.5 seconds before the end press event. These windows were used to analyze the neuronal firing patterns, correlations and information encoded during the task (Figure 1B). While there is considerable overlap in these time windows, the important consideration is not the length of the window but the reference point used to align the neural activity. This has the greatest impact on the peak and the latency of the neuronal response, as well as how the information is generated.

Peri-event time histograms (PETH) were obtained in the windows relative to the three events as defined above in a manner similar to our previous work for identifying neuronal activity around footfalls on a treadmill (Kao et al., 2011). The average background firing rate for each neuron was calculated using a pre-chime window of length 1.5 s. The response region was defined by smoothing the PETHs using a zero-phase distortion moving average filter of length 5 bins (25 ms) and noting when the response exceeded the 99% confidence bound of a random Poisson process with the same overall mean as the cell’s mean firing rate. The response of the neuron to a particular event was considered significant if at least three bins in the unsmoothed response window crossed the upper limit of the 99% confidence interval of the background average. Only cells with a significant response were further analyzed, using the window that generated the largest response. The following parameters were extracted from the unsmoothed PETHs: (a) Response Magnitude (RM): sum of the spikes in all the bins in the response window, divided by the total number of trials after subtracting the average background activity. (b) Peak Response (PR): the bin with the maximum number of spikes divided by the total number of trials after subtracting the average background activity. (c) First bin latency (FBL): The latency of the first bin that crosses the upper bound of the 99% confidence interval described above. (d) Last bin latency (LBL): The latency of the last bin that exceeds the confidence interval. (e) Peak latency (PL): The latency of the peak bin. Differences in these parameters were assessed using a two-way analysis of variance (ANOVA) with two factors; event (levels: chime, start press and end press) and experimental mode (levels: BC mode and NC mode). Post-hoc analysis was done using a Tukey HSD post-hoc test. *Passive sensory responses* were evaluated by lightly anesthetizing the animal and touching the cutaneous surface with a probe identical to the methods outlined in previous studies in our lab [52].

*Responses to footfalls on the treadmill* were calculated by having the animal walk on a motorized treadmill, and evaluating the time of footfalls on the treadmill using videotape. The response of neurons around the time of footfalls was found using methods identical to those outlined in our previous experiments [54].

*Correlations* between neuronal firing and kinematic parameters of behavior (amplitude, reaction time, peak velocity and duration) were measured in a 1500 ms window. Pearson’s linear correlation coefficient between each of the four kinematic parameters and the spike count in the response window was found for each neuron. All the neurons which were recorded simultaneously in an ensemble on a particular recording day having a significant linear correlation with any parameter were then used to fit a multiple linear regression model. The coefficient of determination R^2^ was used to evaluate the model fit and differences were assessed using a two way ANOVA. The first factor was mode with two levels: BC and NC and the second factor kinematic parameter with four levels: amplitude, reaction time, peak velocity and duration.

*The PETH-based classification method* was used to quantify the amount of information about the movement that can be decoded from the activity of the population of neurons. For each movement parameter, the trials were sorted based on the magnitude of parameter, and then partitioned into two groups containing the upper 30% and lower 30% trials. This was optimally chosen for the current range of movements to classify between movements of higher and lower magnitude. *The PETH based classifier* was used to classify between the upper and lower values of each kinematic parameter. The information encoded by the neuronal ensemble about the movement was evaluated by applying Shannon’s information formula to the classification performance. The PETHs were aligned to the three events during the task. The length of the time windows were increased in increments of 100 ms until the window length was 1500 ms as described below:

1. Starting from **chime going forward** upto 1500 ms after chime.
2. Starting from **1500 ms after chime going backward** upto chime.
3. Starting from **end-press going backward** upto 1500 ms before end-press.
4. Starting from **1500 ms before end-press going forward** upto end-press.
5. Starting from **start press**, the window was incremented by 50 ms in both directions till the window length was 1500 ms.

#### Bootstrapping the information

The final value of information reported was obtained after subtracting the value of the bootstrapped information obtained by randomly pairing trials and responses and using the same classification method to obtain the bootstrapped information [55].

#### Dimension reduction using principal component analysis

The spikes in the bins of the PETHs described above were transformed using Principal Component Analysis and the minimum number of principal components sufficient to get the peak value of information obtained using all the PCs was determined by applying the PETH based method repeatedly as the number of PCs used was decreased.

#### Decoding the trajectory using Wiener filter

In order to decode the position of the hindlimb from the neural activity a Wiener filter was used. The position and velocity of the hindlimb was modeled as a weighted sum of the spiking activity of all the neurons recorded simultaneously from the hindlimb sensory motor cortex. The basic form of the equation describing this is
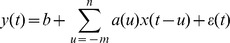
where y(t) is the vector of the movement parameters decoded at time t, x(t-u) is a vector with the neuronal firing rates at time t with a lag u, a(u) is the vector of weights required to fit x(t) to y(t) as a function of the lag, b is the y intercept in this regression and is a constant, 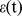 is the residual error term. Neuronal firing rates were sampled using 50 ms bins, a lag of 5 bins was introduced between the neuronal firing and predicted kinematic parameter. Models were trained with approximately 50% of the data and tested on the remaining 50% in cross validation procedure. Pearson’s correlation coefficient, R, between the tested signal (hindlimb position) and the predicted output was calculated. One way ANOVA with factor, method used for reconstruction (with and without feedback of the actual position from one lag back) was used to determine if there were any differences in the decoding ability based on which method was employed.

## Supporting Information

Figure S1 Data showing absolute time for recovery of information after TX. The value of the information decoded about the motor program to press is reported on days 10 (two animals), 30 (four animals), 60 (two animals) and 150 (1 animal) days after TX for 4 animals that were recorded post TX. Two animals were re-introduced into the task 30 days after TX (WB013 and WB020). One of these animals was recorded until day 60 after TX; the other animal was recorded until day 150 after TX. The remaining two animals (WB009) and WB010) were re-introduced into the task and recorded until performance reached behavioral control. The time course for recovery of the information was 10–15 days regardless of when the animals were reintroduced into the task.
(TIF)
